# Plant identity and shallow soil moisture are primary drivers of stomatal conductance in the savannas of Kruger National Park

**DOI:** 10.1371/journal.pone.0191396

**Published:** 2018-01-26

**Authors:** Rebecca L. Tobin, Andrew Kulmatiski

**Affiliations:** Department of Wildland Resources and Ecology Center, Utah State University, Logan, Utah, United States of America; Unesp, BRAZIL

## Abstract

Our goal was to describe stomatal conductance (g_s_) and the site-scale environmental parameters that best predict g_s_ in Kruger National Park (KNP), South Africa. Dominant grass and woody species were measured over two growing seasons in each of four study sites that represented the natural factorial combination of mean annual precipitation [wet (750 mm) or dry (450 mm)] and soil type (clay or sand) found in KNP. A machine-learning (random forest) model was used to describe g_s_ as a function of plant type (species or functional group) and site-level environmental parameters (CO_2_, season, shortwave radiation, soil type, soil moisture, time of day, vapor pressure deficit and wind speed). The model explained 58% of the variance among 6,850 g_s_ measurements. Species, or plant functional group, and shallow (0–20 cm) soil moisture had the greatest effect on g_s_. Atmospheric drivers and soil type were less important. When parameterized with three years of observed environmental data, the model estimated mean daytime growing season g_s_ as 68 and 157 mmol m^-2^ sec^-1^ for grasses and woody plants, respectively. The model produced here could, for example, be used to estimate g_s_ and evapotranspiration in KNP under varying climate conditions. Results from this field-based study highlight the role of species identity and shallow soil moisture as primary drivers of g_s_ in savanna ecosystems of KNP.

## Introduction

Stomatal conductance (g_s_) is a measure of gas exchange between plants and the atmosphere. Stomatal conductance is an important component of water and CO_2_ cycles at both local and global scales [1,2,3]. For example, the g_s_ models developed by Jarvis [4] (including subsequent Jarvis-type models [5] and Ball, Berry, and Woodrow models [6]) are widely-used to estimate canopy-level processes and global circulation of atmospheric gasses [7,8]. These models of g_s_ are empirical, but have been integrated with more mechanistic models that are founded on a sound understanding of the cellular and leaf-level drivers of g_s_, such as CO_2_ concentration, irradiance, and vapor pressure deficit (VPD) [2,7,8,9,10].

While g_s_ is fairly well understood at the cellular and leaf level, it is less understood at the landscape scale where factors such as species identity, soil moisture, disease, plant age and interactions among these factors can affect g_s_ [1,2,5,9,11]. One central problem for understanding landscape-scale g_s_ is that the field-based datasets used to build and validate models are typically small (*i*.*e*., several 100 measurements) or are inferred from ecosystem flux or sapflux measurements [10,12,13,14]. Small g_s_ datasets are unlikely to capture the variability in g_s_ that occurs among species, within canopies, and over daily and seasonal time-scales in response to environmental drivers [13,15,16,17]. Without large field-based datasets, it is difficult to know which parameters are of primary importance to g_s_ under field conditions [10,14,18,19].

There is a particular need for understanding g_s_ in savanna ecosystems [16,20]. Savannas are defined by codominance of grasses and woody plants that may vary widely in abundance and also in their g_s_ responses to environmental drivers [10,12,21]. Further, because savannas are geographically extensive and because savanna g_s_ responses are likely to be sensitive to changes in climate and human management (*e*.*g*., grazing and fire suppression), these systems are likely to have important impacts on global scale biosphere-atmosphere feedbacks [1,22].

As in most ecosystems, g_s_ in savannas has been observed to decrease with VPD and soil moisture [23,24], have a saturating increase with light intensity [10,23] and a unimodal response to temperature [12]. However, soil moisture in these semi-arid systems may have an overriding effect on g_s_ [10,12,25]. Further, different precipitation regimes and seasonal responses to these precipitation regimes have been found to affect how plants exercise stomatal control [26,27]. Species identity may also be critical to understanding g_s_ in savannas. Grasses are likely to have shallower rooting profiles, making them more sensitive to within-season droughts [28,29,30]. Similarly, g_s_ of grasses using the C4 photosynthetic pathway have been found to be highly sensitive to water stress [31] (though at least one study has failed to detect differences between C4 grass and C3 woody plants in a West African savanna) [23]. Further, grasses are likely to realize different environments than their woody plant counterparts because short, dense grass canopies are decoupled from the above-canopy atmosphere and because grasses occur both underneath and between tree canopies [32].

While leaf-level models of g_s_ focus on the roles of [CO_2_] and VPD [10], we predicted that variation in landscape-level g_s_ in a sub-tropical savanna will be best explained by soil water availability and plant type. This is because soils often dry during the growing season in semi-arid systems and dry soils can induce stomatal closure regardless of [CO_2_] and VPD. We predicted that species effects will be important because grasses and woody plants co-dominate savannas and differ in their rooting patterns [30,33].

The overarching goal of this study was to describe site-scale g_s_ in the savannas of Kruger National Park (KNP), South Africa. Because the 19,485 km^2^ KNP encompasses a range of precipitation regimes [from 450 to 750 mm mean annual precipitation (MAP)] and soil types (clay and sand), measurements were made in four sites that represent the natural factorial combination of precipitation (wet or dry) and soil type (clay or sand) found in KNP [34]. More specifically, our objectives were to 1) develop a dataset large enough to describe g_s_ across the four dominant bioclimatic areas found in KNP, 2) use the dataset to build a statistical model of g_s_ as a function of environmental drivers and 3) use the model to produce continuous estimates of grass and woody plant g_s_ using three years of observed environmental data.

## Materials and methods

### Study site

This study was performed in KNP and was approved by the South African National Parks (project registration number 213896412). KNP is located in north-east South Africa between 30.9–32.0 °E and 22.3–25.5 °S. It is characterized by hot, wet summers (October through May) and cool, dry winters (June through September) [34]. A rainfall gradient from north to south in the park produces a range of mean annual precipitation (MAP) from 450 mm to 750 mm yr^-1^, which is representative of a large proportion of precipitation-limited savannas [34,35,36]. Most of the eastern half of KNP is underlain by basaltic rock that weathers into nutrient-rich, clay-rich soils, while the western half is underlain by granitic rock that weathers into nutrient-poor, sandy soils [37]. Four study sites were selected that represented a natural factorial combination of precipitation regimes (“wet” or “dry”) and soil texture (“sand” or “clay”; Table 1) [38] found in the park. The four sites occur on very gently rolling to flat terrain. Water infiltration occurs through the top 1 m of soil within a few days of large, mid-season precipitation events at all sites, but little water infiltrates below this rooting zone [30,39,40]. Even if total annual precipitation occurred as a single event, it would be expected to saturate soils to 3.0 to 3.7 m in the different sites.

**Table 1:** Precipitation regimes, soil types and sampling seasons corresponding to the four study sites in Kruger National Park, South Africa. Growing seasons occur from October through May and are referred to by the year during which the growing season ends.

Site Name	Precipitation Regime	Soil Type	Season Sampled
Letaba	dry (450 MAP*)	clay (calcareous shallow clay)	2010, 2013
Phalaborwa	dry (475 MAP)	sand (coarse fersiallitic sand)	2011, 2012
Lower Sabie	wet (730 MAP)	clay (pedocutanic clay)	2011, 2013
Pretoriuskop	wet (750 MAP)	sand (coarse fersiallitic sand)	2008, 2009

*MAP = Mean annual precipitation in mm.

The four study sites are typically dominated by one to three woody plant species and one to three grass species [34]. Dominant C4 grasses at the four sites were *Bothriochloa radicans* (Lehm) A. Camus, *Schmidtia pappophoroides* (Steud.), *Urochloa mosambicensis* (Hack) Dandy and *Cenchrus ciliaris* L. at the dry/clay, dry/sand, wet/clay and wet/sand sites, respectively. Dominant woody plants were *Colophospermum mopane* (Kirk ex Benth.) at the dry/clay and dry/sand sites, a mix of *Securinega virosa* (Roxb.), *Strychnos spinosa* (Lam.) and *Dalbergia melanoxylon* (Guill. and Perr.) at the wet/clay site and *Sclerocarya birrea* and (A. Rich) and *Terminalia sericea* (Burch ex. DC) at the wet/sand site.

### Study design

#### Stomatal conductance measurements

The four sites were each sampled during two different seasons (Table 1). During each season, measurements were made over two to three days during two early- (November, December), two mid- (January, February) and two late-season (March, April) sampling periods. As a result, g_s_ was measured on 24 to 36 different days at each site. During each sampling day, one to four people with a list of target species and a steady-state porometer (Decagon Devices, SC-1) [41], would walk along a transect until they encountered one of four to nine target species (Table 2). The number of target species was selected so that sampled species represented 80% of total leaf area at a site. Leaf area was determined in separate research using 96, 1-m^2^ quadrats [30,33]. Measurements were taken throughout the plant canopy. For grasses, measurements were made near the end and near the base of leaves on both adaxial and abaxial surfaces. For woody plants, only abaxial measurements were detectable. Woody plant leaves were sampled from top, middle, bottom and inner canopy positions roughly proportionate to their abundance. Target species would be sampled within 15-minute increments of either consistently clear or consistently overcast conditions. After collecting samples from the target species for a site, additional time in the 15-minute increments was used to take extra samples from the most dominant species. Measurements were made to be roughly representative of species abundance and at the end of the study the number of g_s_ measurements per species was positively correlated with leaf area by species (R^2^ = 0.66). Samples were taken between sunrise and sunset, though few measurements were taken at dawn and dusk due to safety concerns of working in the park.

**Table 2:** Species, plant functional type and sample sizes from each study site. DC = dry / clay (Letaba), DS = dry / sand (Phalaborwa), WC = wet / clay (Lower Sabie), WS = wet / sand (Pretoriuskop). Numbers in parentheses indicate sample size. Remaining sampled species listed in S1 Table.

Species	Plant type	Sample Size by Site
*Bothriocloa radicans*	Grass	DC (456), DS (12), WC (60)
*Cenchrus ciliaris*	Grass	WS (458)
*Loudetia simplex*	Grass	WS (134)
*Panicum spp*.	Grass	DC (11), WC (234)
*Schmidtia pappophoroides*	Grass	DS (71)
*Urochloa mosambicensis*	Grass	DC (25), DS (76), WC (245)
*Acacia nigrescens*	Woody	DS (33), WC (208)
*Colophospermum mopane*	Woody	DC (843), DS (178)
*Combretum apiculatum*	Woody	DC (12), DS (102)
*Combretum imberbe*	Woody	DS (36), WC (68)
*Dichrostachys cinerea*	Woody	DS (106), WS (749), WC (402)
*Grewia bicolour*	Woody	DS (12), WC (109)
*Lonchocarpus capassa*	Woody	DS (15), WS (3), WC (107)
*Sclerocarya birrea*	Woody	DC (2), DS (24), WS (148), WC (1)
*Securinega virosa*	Woody	DS (128), WC (298)
*Terminalia sericea*	Woody	DS (1), WS (797)
*Ximenia caffra*	Woody	WS (106)

#### Environmental parameters

Temperature, relative humidity (215L; Campbell Scientific, UT, USA), wind speed (014A cup anemometer; MetOne, OR, USA) and shortwave radiation (SP-110; Apogee Instruments, UT, USA) were measured at 1 m and 2 m heights. Measurements at 1 m and 2 m were used for models of grass and tree g_s_, respectively. Precipitation (Texas Instruments TE-525; Texas Instruments, TX, USA) and soil water potential were measured at one location at each site. All measurements were recorded hourly at each site on Campbell Scientific CR1000 dataloggers. Air temperature and relative humidity were used to calculate VPD. Atmospheric CO_2_ measurements were provided by a flux tower near Skukuza, which was 32 to 136 km from the study sites [42,43]. Inasmuch, CO_2_ data provided inference to broad, regional scale patterns and not site-specific patterns of CO_2_ concentrations. The CO_2_ sensor was located eight meters above most of the canopy and six meters above the tallest trees in the flux tower fetch [42,43]. Heat dissipation sensors (Campbell 229L; Campbell Scientific, UT, USA) were used to calculate soil water potential hourly for 0–20 cm, 0–50 cm, 20–50 cm, and 50–150 cm depths for each site. Heat dissipation sensors were calibrated individually prior to installation [30,44].

#### Data analyses

Because we sampled across four distinct sites, we first used a one-way analysis of variance to test for differences in mean g_s_ values among sites for each plant functional type (woody plants and grasses). To meet assumptions of normality, g_s_ values were log-transformed. Because sample sizes differed among sites, Type III sum of squares were used and pairwise comparisons were examined using the Tukey test. Species with less than 100 measurements in the dataset were excluded.

#### Random forest modeling

We used a machine-learning model to 1) describe the relationship between environmental parameters and g_s_, 2) build a predictive model of site-scale g_s_ and 3) estimate site-scale g_s_ using three years of environmental data. RF models provide the mode or mean of many different classification or regression trees, respectively, and correct for the tendency for decision trees to overfit data [45]. RF modeling reveals nonlinear relationships and complex interactions in ecological data that may be missed by other statistical methods [45].

RF models were used to describe the effect of the following parameters on g_s_: atmospheric CO_2_ (ppm), precipitation regime (wet or dry), shortwave radiation (μmol m^-2^ sec^-1^), soil moisture (soil water potential, MPa), soil type (clay or sand), species identity, time-of-day (hour), time-of-season (early, mid, late), VPD (kPa) and wind speed (m sec^-1^). Because g_s_ can vary widely with plant functional type, and because it is reasonable to expect that grasses and woody plants may respond differently to environmental drivers [46] and because savannas show wide variations in woody plant cover [34,35], g_s_ was modeled separately for each functional group. Measurements of g_s_ were paired with meteorological and soil measurements from the closest recorded time step. Where meteorological data was missing for a time step, data were interpolated by correlating and adjusting data from the nearest weather station using a linear equation. Less than 10% of the meteorological data were interpolated. To test for potential lag effects in the response of g_s_ to environmental conditions, the 3-hour averages of air temperature, relative humidity, VPD, wind speed, and shortwave radiation, the 3-hour, 24-hour, and 7-day averages of each soil moisture depth, and the 24-hour sum of precipitation were calculated and used in the model.

Although highly-correlated predictors do not affect RF variable importance [45], to simplify interpretation of variable relationships, correlation matrices for groups of related predictor variables were used to test for multicollinearity [45]. Air temperature, precipitation, relative humidity, soil moisture, shortwave radiation, VPD and wind speed were evaluated using Spearman correlation. Where two predictors were highly correlated, the predictor with the greatest adjusted-squared-deviance explained by a generalized linear model (GLMs; linear + quadratic, family = Gaussian) was used in the RF model [45]. When highly-correlated predictors explained similar amounts of variance in g_s_ (difference of less than 2%), separate RF models were created and the variable that explained the most variance was selected.

To estimate the effect that each variable has on model output (i.e., variable importance), values for each variable were randomized, one at a time, and the prediction error in models with randomized and non-randomized values calculated [45,47]. Large prediction error (*i*.*e*., variable importance; VIMP) values indicated that specifying the variables incorrectly increased prediction error; therefore, variables with large VIMP values are more important [47].

Relationships between g_s_ and environmental parameters were characterized with risk-adjusted partial dependence plots [47]. Partial dependence refers to the dependence of the response variable, in this case g_s_, on one predictor variable [45]. The plots were created by averaging the effects of the remaining environmental parameters and predicting how the response variable changes with the predictor of interest alone [45].

Model estimates of g_s_ were generated using site-specific data from the 2009/2010, 2010/2011 and 2011/2012 growing seasons. The data were collected and prepared using the same instrumentation and methods as the data used to build the RF models. The data were then run through the RF models using the predict function in the randomForestSRC package [45]. Model estimates were generated separately for each study species present at a site. Average g_s_ by species (weighted by species abundance) were produced for each plant functional type in each study site and for the entire park. Statistical analyses were performed in RStudio [48]. All RF models and predictions were developed using the R package randomForestSRC [48] and all model visualizations were created using the ggRandomForests package [47].

## Results

**Fig 1:**
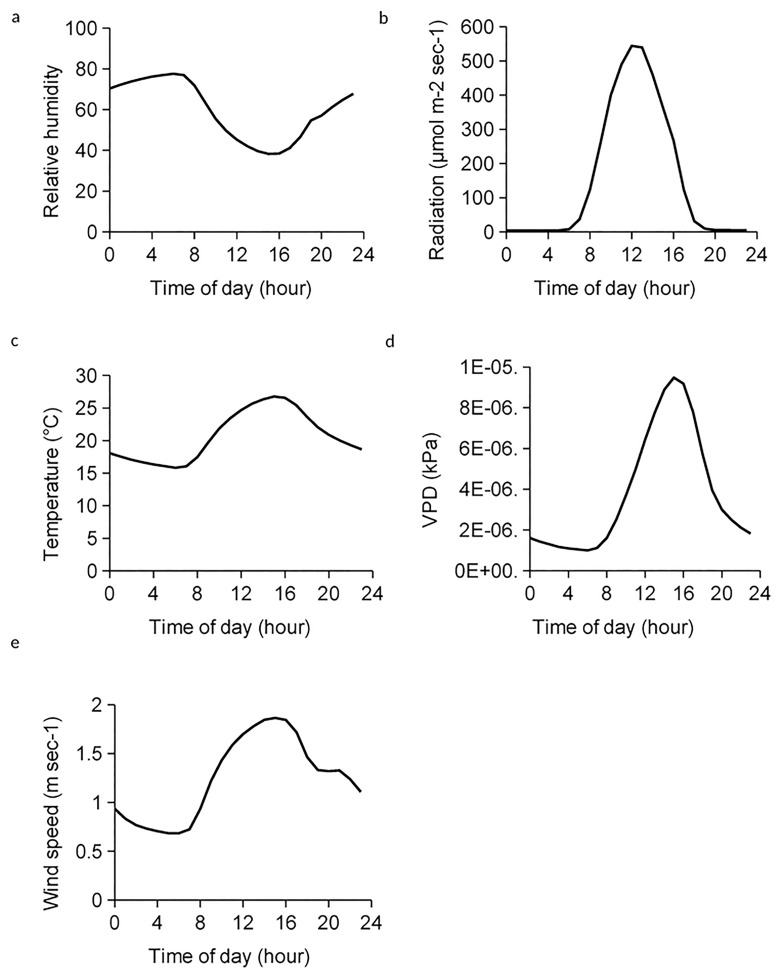
Average daily values of (a) relative humidity, (b) shortwave radiation, (c) temperature, (d) vapor pressure deficit (VPD), and (e) wind speed over two years at the Pretorioskop study site, Kruger National Park, South Africa.

We present average diurnal environmental conditions from the Pretorioskop site to provide general context of site conditions (Fig 1). Temperature, radiation, VPD, and wind speed all reached maximum average daily values between 16:00 and 17:00 hours at 26.8° C, 1491 μmol m^-2^ sec^-1^, 9.18 kPa and 1.84 m sec^-1^. Relative humidity realized an average daily minimum at 16:00 hours at 38.3%.

### Observed g_s_

**Fig 2:**
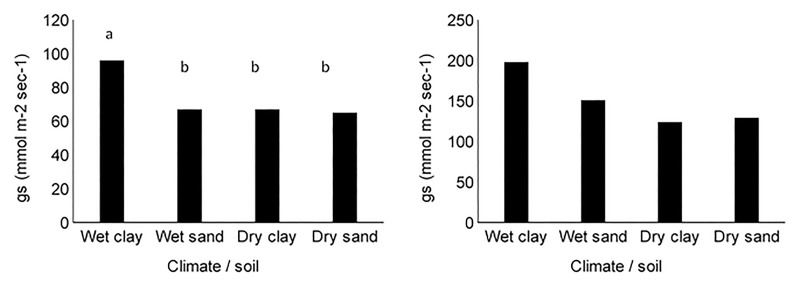
Stomatal conductance (gs) of grasses (a) and woody plants (b) measured over five years in four sites in Kruger Park that varied in precipitation regime from 400 mm mean annual precipitation (MAP; Dry) to 750 mm MAP (Wet) on either clay or sand soils.

Over the six years of the study, 8,510 g_s_ measurements were made across the four sites (Table 2). However, 1,080 of these measurements were from species that were measured less than 100 times and were excluded from analyses. Mean observed daytime g_s_ was 75 ± 1 mmol m^-2^ s^-1^ for grasses and 155 ± 2 mmol m^-2^ s^-1^ for woody plants. Forb g_s_ was measured for related research and was 142 ± 5 mmol m^-2^ s^-1^. Because forbs represented < 10% of leaf area and sample size (n = 580), they were excluded from further analyses. Mean observed g_s_ for grasses was greater in the wet/clay site than the wet/sand, dry/clay or dry/sand sites (Fig 2; *F* = 28.40, p < 0.001). Mean observed g_s_ for woody plants was greater in both wet sites than the dry sites and greater in the wet/clay site than the wet/sand sand site and the wet/sand site was greater than either the dry/clay or dry/sand sites (Fig 2; *F* = 77.30, *p* < 0.001).

### Random forest

With measurements from uncommon species removed, RF modeling was performed on a dataset with 6,850 measurements from common grass and woody plant species. The best RF model for this dataset explained 58% of variance and included, in descending order of importance, species, 24-hour shallow (0–20 cm) soil moisture, 24-hour deep (50–150 cm) soil moisture, 3-hour shortwave radiation, 3-hour VPD, 3-hour wind speed, atmospheric CO_2_, time-of-season, time-of-day, precipitation regime, and soil type. This model was used to estimate g_s_ across three growing seasons (see below). When species was replaced with plant functional type, the percent variance explained by the model decreased to 51% but the order of variable importance remained the same. When neither species nor plant functional type was included in the model, percent variance explained decreased to 40%. Percent variance explained for the grass dataset with and without species was 27% and 21%, respectively. Percent variance explained for the woody g_s_ dataset with and without species was 54% and 45%, respectively. Remaining analyses were performed separately for grasses and woody plants. Due to multicollinearity, precipitation, relative humidity, 0–50 cm soil moisture, 20–50 cm soil moisture, seven-day soil moisture, three-hour soil moisture, and temperature, were not included in subsequent analyses.

#### Variable importance

**Fig 3:**
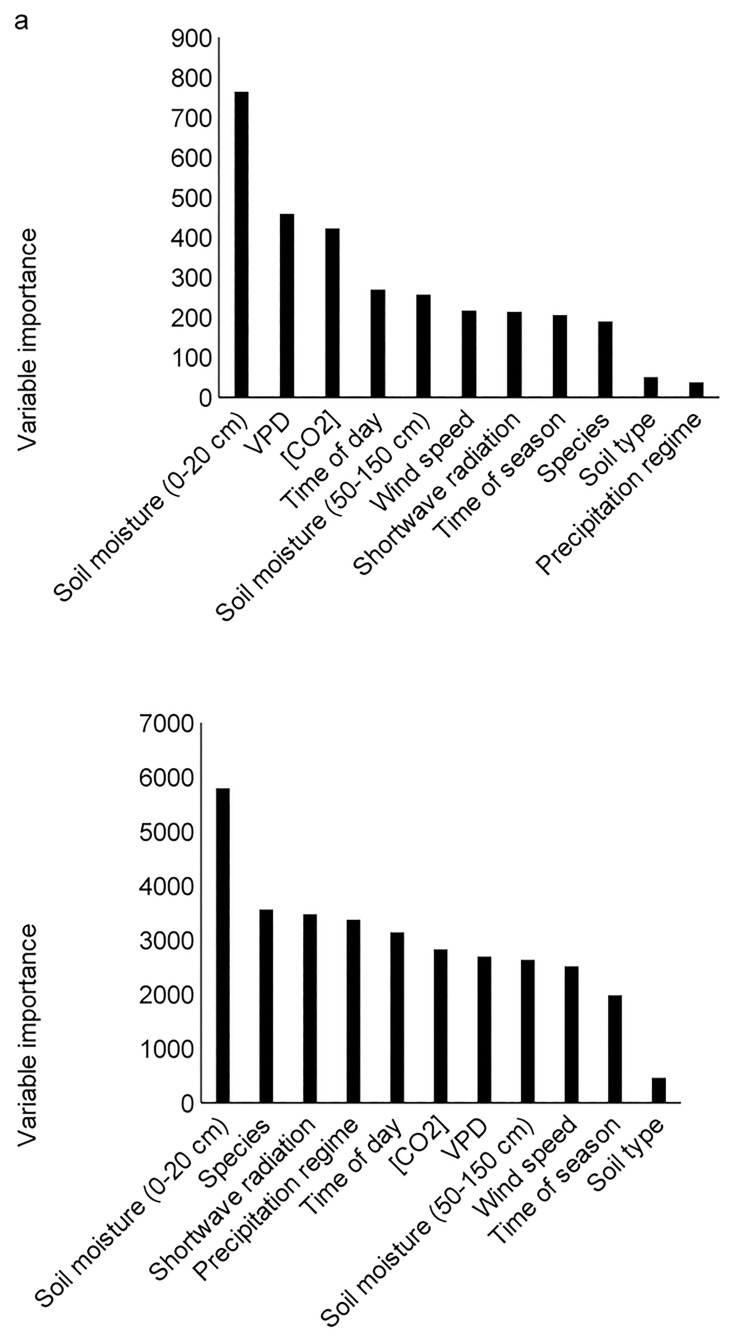
Variable importance in random forest models of stomatal conductance for (a) grasses and (b) woody plants. For both grasses and woody plants shallow (0–20 cm) soil moisture and soil type (*i*.*e*., clay or sand) were the most and least important variables describing g_s_, respectively. Variable importance is the difference in prediction error before and after a predictor variable is randomly permutated. Large variable importance values indicate that specifying the variables incorrectly increases prediction error. See text for further variable descriptions.

For both grasses and woody plants, shallow (0–20 cm) soil moisture was the most important predictor of g_s_, explaining 7 and 10% of the variation in the grass and woody plant datasets, respectively (Fig 3). The remaining variables differed in importance between grasses and woody plants. For grasses, in descending order of importance: VPD, atmospheric CO_2_, species, wind speed, shortwave radiation, deep (50–150 cm) soil moisture, time-of-season, time-of-day, soil type, and precipitation regime explained variance in g_s_ (Fig 3a). For woody plants, in descending order of importance: shortwave radiation, precipitation regime, species, CO_2_, VPD, wind speed, deep soil moisture (50–150 cm), time-of-season, time-of-day, and soil type explained variation in g_s_ (Fig 3b).

**Fig 4:**
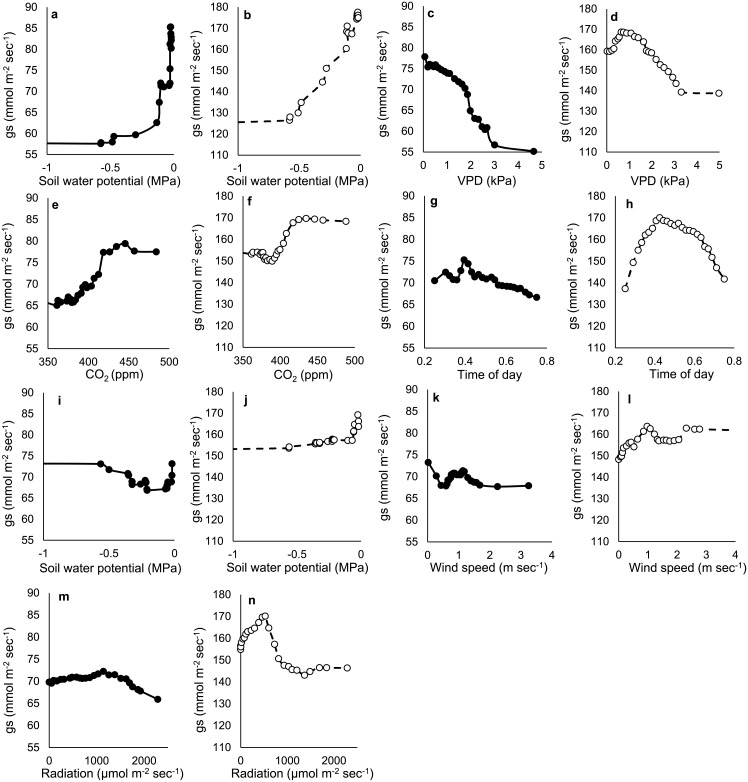
Partial dependence of grass and woody plant stomatal conductance (g_s_) on environmental drivers. Values shown are predicted grass and woody plant g_s_ as a function of each variable when all other variables are held at their mean value. Drivers listed in descending order of importance to grass g_s_: shallow soil moisture (0–20 cm; a and b), vapor pressure deficit (VPD; c and d), atmospheric CO_2_ (e and f), time of day (g and h), deep soil moisture (50–150 cm; i and j), wind speed (k and l), and shortwave radiation (m and n). Note that axis values are different for grasses and woody plants.

In descending order of importance of continuous variables, grass g_s_ increased 51% with shallow soil moisture, decreased 29% with VPD, increased 22% with CO_2_, decreased 16% with shortwave radiation above ~500 μmol m^-2^ sec^-1^, increased 11% from midday to dusk, and showed less than a 10% change with deep soil moisture, shortwave radiation and wind speed (Fig 4; Percentages here represent the difference between maximum and minimum values); Woody g_s_ increased 47% with shallow soil moisture, decreased 18% with VPD values above 0.6 kPa, increased 17% with deep soil moisture, decreased 14% with shortwave radiation values above 500 μmol m^-2^ s^-1^, increased 13% with CO_2_ and increased 10% with wind speed (Fig 4). G_s_ differed by more than 10% for only one categorical value: woody g_s_ was 16% greater in wet sites than dry sites (S1 Fig).

#### Random forest model estimates of g_s_

Mean daytime g_s_ across three years at the four study sites was 68 and 157 mmol m^-2^ s^-1^ for grasses and woody plants, respectively. Mean estimated g_s_ was notably greater in the wet/sand site for grasses (78 mmol m^-2^ s^-1^) and woody plants (253 mmol m^-2^ s^-1^) than other sites. Mean estimated grass g_s_ was 68, 65 and 62 mmol m^-2^ s^-1^ in wet/clay, dry/sand and dry/clay sites, respectively. Mean estimated g_s_ for woody plants was 153, 135, and 131 mmol m^-2^ s^-1^ in wet/clay, dry/sand and dry/clay sites, respectively (S3 Fig). The coefficient of variation was 12% among the five dominant grass species and 29% among the 11 dominant woody species (S2 Fig). The larger variation among woody plants reflected large g_s_ values for *Terminalia sericea* (258 mmol m^-2^ s^-1^) and *Ximenia caffra* (256 mmol m^-2^ s^-1^), which were found primarily in the wet/sand site (Table 2; S3 Fig).

## Discussion

Results from this field-collected dataset highlight the importance of plant identity (functional type or species) and shallow soil moisture as primary drivers of g_s_ in the tropical and sub-tropical savannas of Kruger National Park. Consistent with previous research, our analyses detected a negative g_s_ response to VPD [49]. However, for several other meteorological variables, such as atmospheric CO_2_ and shortwave radiation, the g_s_ response found here was less consistent with previous research [7,50,51,52,53]. The data and model reported here, therefore, provide a perspective on site-scale values and drivers of g_s_ that differs from many laboratory-based approaches and the difference appears likely to be driven by the over-riding importance of soil moisture on g_s_ in these semi-arid ecosystems. While results differed from laboratory-based, leaf level measurements that emphasize the role of CO_2_ and VPD, our results are consistent with canopy-level estimates of ET derived from a flux tower in Kruger Park [43].

Plant identity had the greatest effect on g_s_. In part, this reflected the fact that woody plant g_s_ was twice as large as grass g_s_; although, even within grass and woody plant functional groups, species explained a large portion of variation in g_s_. Much of the ‘species’ effect was caused by large values for the woody plants *Terminalia sericea* and *Xemenia caffra*. Values for these two species were 79% greater than the mean g_s_ for the other nine common woody species. The response of *T*. *sericea*, in particular, is not trivial because this species is common and often dominant in southern Africa and related species are common in Australia. Because much of the variation among species was caused by grasses vs. woody plants and 2 of the 11 woody species, it is possible that identifying and incorporating information on plant functional type and a small subset of species with large g_s_ values may significantly improve regional models of g_s_ [20,54,55].

Values of g_s_ were surprisingly similar among sites. Mean g_s_ values estimated across three growing seasons differed by only 3 to 26% among sites with the notable exception that woody g_s_ values were 65 to 93% greater in the wet/sand than any other site. This reflected the dominance of the fast-transpiring *T*. *sericea* in the wet/sand site. In contrast, soil type had little effect on g_s_. This was surprising but likely occurred because our use of soil water potential values controlled for soil texture effects on soil water availability. Results suggested that g_s_ is likely to be quite similar among a wide range of precipitation regimes and soil types, but that g_s_ models that account for changes in species composition will produce better predictions than those that do not. It is important to note that if species or soil moisture data were not available, soil type would have been likely to help explain variance in g_s_, however, our results indicate that these ‘soil type’ effects are caused mostly by soil type effects on species composition and soil water availability.

**Fig 5:**
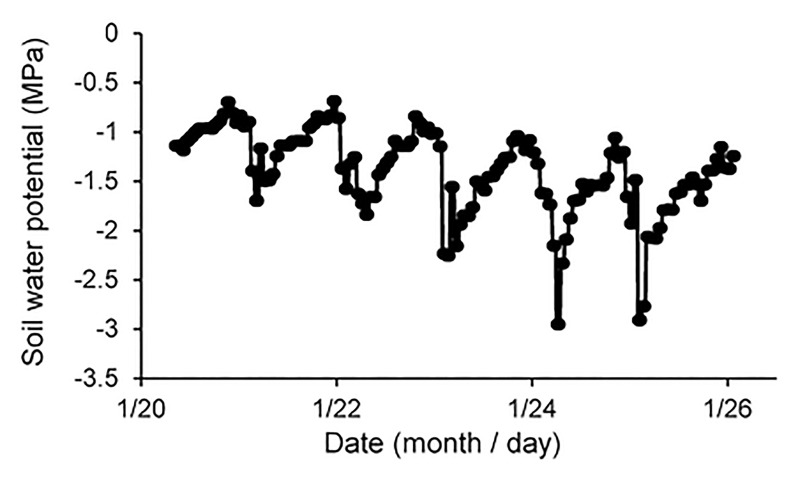
An example of diurnal variation in soil water potential (MPa) at 20 cm over six days at the Letaba study site, 2010. Soil water potentials were greatest each day between 8 am and 11 am and smallest each day between 2 pm and 7 pm. Consistent with hydraulic redistribution, this indicated that shallow soils gradually became ‘wet’ each night and quickly dried between roughly 9 am and 5 pm.

After species and functional group, soil moisture was the most important driver of landscape-scale g_s_. It is important to note that soil moisture was estimated from measurements of soil water potential and not soil water content. Soil water potential measurements provide a better estimate of soil water availability than soil water content because soil water potential controls for the effects of soil texture. Shallow soil moisture (*i*.*e*., soil water potential in the 0–20 cm depths), in particular, was the most important driver of g_s_ for both grasses and woody plants. Results indicated a fairly conservative stomatal control response with both grasses and woody plants decreasing g_s_ roughly 50% between fairly moist water potentials of -0.1 to -0.5 MPa [29]. Midday leaf water potentials for grasses and woody plants at the Letaba study site have been reported to be roughly -2.5 MPa so stomatal control at water potentials of -0.1 to -0.5 MPa was surprising [30]. Our measurements of soil moisture were averaged across the 0–20 cm depths and across three hour time-steps. It is possible that our averaged, bulk soil measurements overestimated soil water availability in the rhizosphere (Fig 5)[56]. Regardless of the value at which stomatal control occurred, shallow soil water potentials were a dominant driver of both grass and woody plant g_s_ during the six years of sampling in this study. Consistent with recent hydrologic tracer experiments in the park, these results suggest that both grasses and woody plants rely heavily on shallow soil water [30,33].

The dominant role of soil moisture appeared to affect the relationship between g_s_ and other environmental parameters. For example, g_s_ typically decreases with atmospheric CO_2_ [19,21,57], yet, in this study g_s_ increased with atmospheric CO_2_. Atmospheric CO_2_ was highest in the morning and decreased throughout the day [42]. Thus, atmospheric CO_2_ decreased as soil moisture was likely to decrease (Figs 1 and 4) and shortwave radiation and VPD were likely to increase. Each of these diurnal parameter changes were likely to decrease g_s_ at the same time that decreasing CO_2_ concentrations were likely to increase g_s_. Because shallow soil moisture was a primary driver of g_s_, it is likely that soil moisture effects ‘masked’ CO_2_ effects on g_s_. Diurnal variations in soil moisture of this type are common in semi-arid systems and reflect the rapid midday exhaustion of rhizosphere water by root uptake and gradual replenishment of rhizosphere water through soil water flow and hydraulic redistribution [11,58,59]. Regardless of the mechanism, results suggest that CO_2_ fertilization effects seen in some experiments may be difficult to observe in semi-arid savannas due to ‘masking’ effects of other drivers of plant growth such as soil moisture [18,60,61,62].

A similar result was found with shortwave radiation. Values of g_s_ typically increase with radiation to a maximum g_s_ threshold [5,52] but see [32]. Here, however, grass g_s_ showed very little response to shortwave radiation and woody plant g_s_ increased to and then decreased beyond shortwave radiation values of roughly 500 μmol m^-2^ s^-1^. It is possible that g_s_ values in the field decreased as radiation increased because g_s_ was primarily limited by soil moisture and plants were likely to exhaust soil water immediately around their roots in the morning, at the same time that radiation increased [46,50]. In short, it is likely that g_s_ responses to radiation were precluded or ‘masked’ by g_s_ responses to soil moisture. Such midday stomatal depressions are common [58].

Responses of g_s_ to CO_2_ and shortwave radiation highlight both the strengths and weaknesses of observational approaches. Data from this study provide estimates that account for the many interacting factors and emergent properties that affected g_s_ during the years and conditions of this study. This approach showed, for example, the counter-intuitive positive response of g_s_ to CO_2_. The approach, however, is not well designed to isolate the individual effects of the drivers of g_s_ because measurements were made in the field where multiple drivers change simultaneously. Results, therefore, are representative of g_s_ values in field conditions, but cannot provide strong inference to conditions unlike those observed during the study (*i*.*e*., extreme conditions associated with climate change).

In contrast to responses to shortwave radiation and CO_2_, g_s_ decreased with VPD as would be expected from laboratory-based studies. This effect was the second most important driver for grasses and also important for woody plants: g_s_ decreased 29% and 21% with increasing VPD for grasses and woody plants, respectively. Our detection of a VPD effect was likely due, in part, to the fact that VPD and rhizosphere soil moisture are both likely to decrease g_s_ through the day.

This study highlights the difficulties of modeling g_s_ at the site- to landscape-scale [14]. Despite large sample sizes, the dataset was highly variable, and RF models explained only a modest proportion of variance, particularly for grasses. It is reasonable to expect that plant-to-plant variation would have explained a large portion of the variation in the dataset; however, averaging measurements over 2-hour increments (instead of 1-hour increments) provided nominal improvements in model power (*i*.*e*., 2% of error). This suggested that plant-to-plant variation explained little of the residual variance.

Variation in our dataset may have been accounted for by leaf-level environmental conditions. As stomatal aperture can change in response to leaf-level conditions, such as interstomatal CO_2_, leaf water potential, and leaf temperature, including these leaf-level parameters may be necessary to explain much of the unexplained variance in the dataset [10]. Indeed, predictions from leaf-level models of g_s_ that incorporate these types of parameters are often strongly correlated with observed values [8,10,12,63]. However, these studies tend to model variation in relatively small datasets, under fairly limited conditions and more importantly require detailed information on photosynthesis that are difficult to collect at large scales [7,10]. An important ‘next step’ for this research will be to compare the quality of model predictions developed here to model predictions from more commonly used semi-empirical models [7,10,64].

This study provides a prioritized list of variables important to landscape g_s_ in this region. Results indicated that plant identity and shallow soil moisture are of greater importance than atmospheric conditions. While incorporating species into global circulation or land surface models may not be practical, it is possible to include plant functional type data or information on common species with very large g_s_ values [64]. Species-level data is more likely to be useful for improving accuracy in canopy or ecosystem-level modeling [28]. This study also provides a model that can be used to produce continuous estimates of g_s_ for the savannas of KNP using basic environmental data [30]. Such estimates are expected to improve evapotranspiration modeling for the savannas of KNP.

Finally, results provided novel insight into fundamental questions about savanna structure and function. The importance of shallow soil moisture to both grasses and woody plants was consistent with a rapidly-developing perspective of niche partitioning for soil water in savannas [30,33,65]. The importance of shallow water and the negative relationship between g_s_ and CO_2_ provided new support for the idea that CO_2_ fertilization effects may be precluded by water limitation in semi-arid systems [60,61,62].

## Supporting information

S1 Fig Partial dependence of grass and woody plant stomatal conductance on soil type and precipitation regime. (DOCX)

S2 Fig Modeled daily and seasonal gs for grasses and woody plants by study site. (DOCX)

S3 Fig Mean daily stomatal conductance (g_s_) for common grass and woody plant species estimated across three growing seasons, Kruger National Park, South Africa. (DOCX)

S1 Table Studied species and their respective common names, families, and growth forms. (DOCX)
