# Supplementary material for: Plant identity and shallow soil moisture are primary drivers of stomatal conductance in the savannas of Kruger National Park
Source: PLoS One. 2018 Jan 26;13(1):e0191396. doi: 10.1371/journal.pone.0191396 (PMC5786297; doi:10.1371/journal.pone.0191396)
Supplement: S1 Fig — (DOCX) [file pone.0191396.s001.docx]

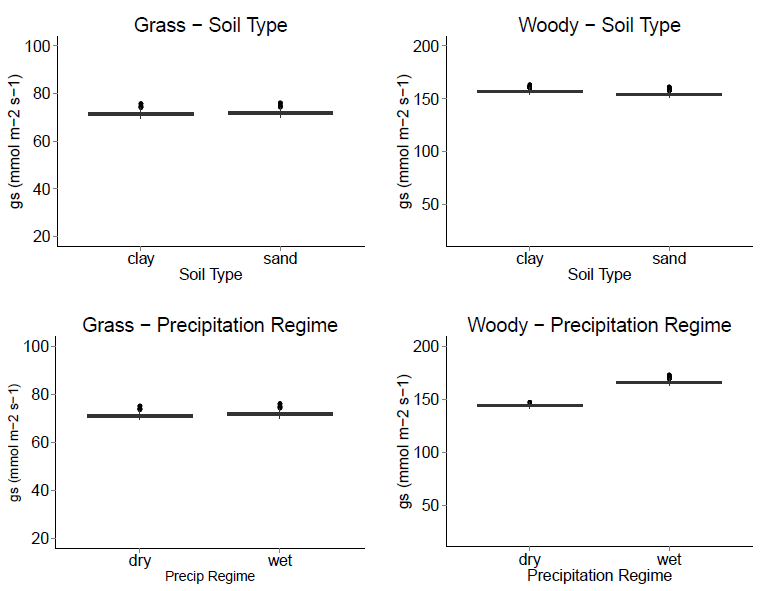


S1 Fig. Partial dependence of grass and woody plant stomatal conductance on soil type and precipitation regime. Partial dependence is determined by averaging the effects of the other predictors and predicting how the response variable changes with the predictor of interest alone. Neither grasses nor woody plants showed a strong gs response to soil type. Woody plants but not grasses had greater gs wet relative to dry sites.
