# Supplementary material for: Plant identity and shallow soil moisture are primary drivers of stomatal conductance in the savannas of Kruger National Park
Source: PLoS One. 2018 Jan 26;13(1):e0191396. doi: 10.1371/journal.pone.0191396 (PMC5786297; doi:10.1371/journal.pone.0191396)
Supplement: S2 Fig — (DOCX) [file pone.0191396.s002.docx]

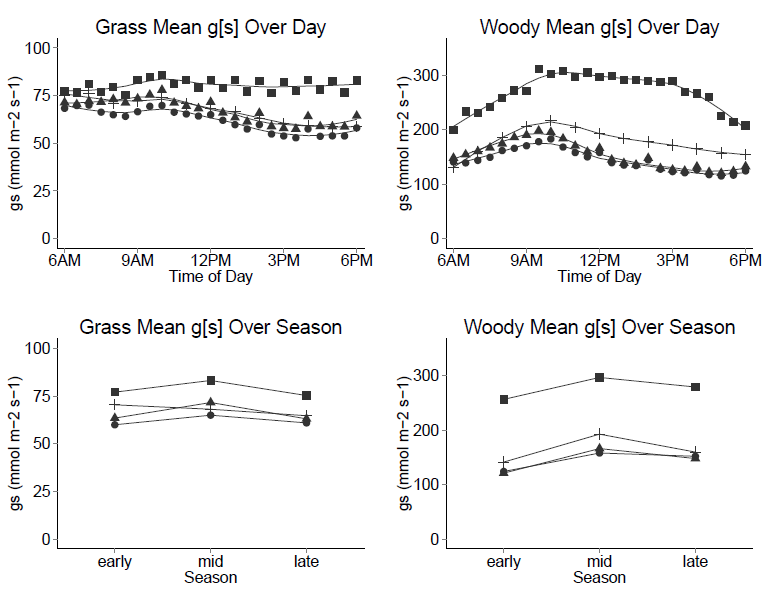


S2 Fig. Modeled daily and seasonal gs for grasses and woody plants by study site. The shape of the points indicates the study site: crosses for the wet/clay site, squares for the wet/sand site, circles for the dry/clay site, and triangles for the dry/sand site. For the plots of daily g_s_, model predictions were averaged for each timestep and mid-season values are shown. For the plots of seasonal g_s_, model predictions were averaged for each season and noon values are shown.
