# Supplementary material for: Plant identity and shallow soil moisture are primary drivers of stomatal conductance in the savannas of Kruger National Park
Source: PLoS One. 2018 Jan 26;13(1):e0191396. doi: 10.1371/journal.pone.0191396 (PMC5786297; doi:10.1371/journal.pone.0191396)
Supplement: S3 Fig — (DOCX) [file pone.0191396.s003.docx]

**S3 Fig. Mean daily stomatal conductance (g_s_) for common grass and woody plant species estimated across three growing seasons, Kruger National Park, South Africa**. G_s_ was measured during six sampling campaigns over two growing seasons in each of four study sites. Measurements were used to create a model of stomatal conductance that was used to produce continuous estimates of g_s_ as function of environmental conditions over three growing seasons.


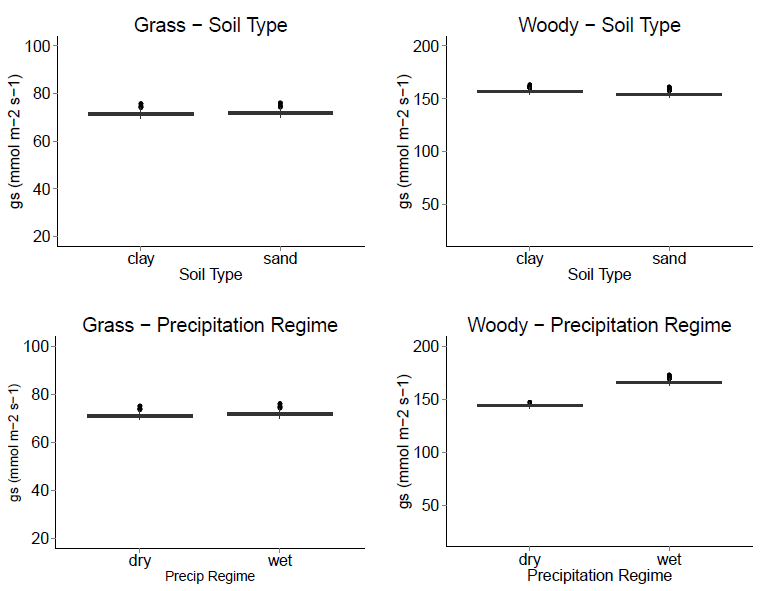


S1 Fig. Partial dependence of grass and woody plant stomatal conductance on soil type and precipitation regime. Partial dependence is determined by averaging the effects of the other predictors and predicting how the response variable changes with the predictor of interest alone. Neither grasses nor woody plants showed a strong gs response to soil type. Woody plants but not grasses had greater gs wet relative to dry sites.
