# Supplementary material for: Plant identity and shallow soil moisture are primary drivers of stomatal conductance in the savannas of Kruger National Park
Source: PLoS One. 2018 Jan 26;13(1):e0191396. doi: 10.1371/journal.pone.0191396 (PMC5786297; doi:10.1371/journal.pone.0191396)
Supplement: S1 Table — (DOCX) [file pone.0191396.s004.docx]

S1 Table. Studied species and their respective common names, families, and growth forms.

| **Species** | **Common/Alt. Names** | **Family** | **Growth Form** |
| --- | --- | --- | --- |
| Acacia nigrescenes | Knobthorn | Fabaceae | Tree |
| Acacia gerrardii | Red thorn | Fabaceae | Tree |
| Acacia tortilis | Umbrella thorn | Fabaceae | Tree |
| Albizia harveyi | Common false thorn | Fabaceae | Tree |
| Lonchocarpus capassa | Apple-leaf | Fabaceae | Tree |
| Aristida sp. |  | Poaceae | Grass |
| Euclea crispa | Blue guarri | Ebenaceae | Tree |
| Bothriochloa radicans | Stinking grass | Poaceae | Grass |
| Cenchrus ciliaris | Buffelgrass, African foxtail grass | Poaceae | Grass |
| Loudetia simplex | Common russet grass | Poaceae | Grass |
| Tragus berteronianus | Carrot seed grass | Poaceae | Grass |
| Combretum apiculatum | Red bushwillow | Combretaceae | Tree |
| Combretum hereroense | Russet bushwillow | Combretaceae | Tree |
| Combretum imberbe | Leadwood | Combretaceae | Tree |
| Gymnosporia buxifolia | Common spike thorn | Celastraceae | Tree |
| Dichrostachys cinerea | Sickle bush | Fabaceae | Shrub |
| Phoenix reclinata | Wild date palm | Arecaceae | Tree |
| Enneapogon conchroides | Nine-awned grass | Poaceae | Grass |
| Euclea divinorum | Magic guarri | Ebenaceae | Shrub/tree |
| Lannea schwinfurthii | False marula | Anacardiaceae | Tree |
| Digitaria erianthra | Common finger grass | Poaceae | Grass |
| Grewia bicolour | White raisin | Malvaceae | Tree |
| Hyperthelia dissolute | Yellow thatching grass | Poaceae | Grass |
| Hyparrhenia filipendula |  | Poaceae | Grass |
| Hyparrhenia hirta | Common thatching grass | Poaceae | Grass |
| Adenium multiflorum | Impala lily | Apocynaceae | Forb |
| Sclerocarya birrea | Marula | Anacardiaceae | Tree |
| Maerua angolensis | Bead bean | Capparaceae | Tree |
| Melinis repens | Natal grass | Poaceae | Grass |
| Strychnos madagascariensis | Black monkey orange | Loganiaceae | Shrub/tree |
| Colophospermum mopane | Mopane | Fabaceae | Shrub/tree |
| Panicum coloratum | Small buffalo grass | Poaceae | Grass |
| Panicum maximum | Guinea grass | Poaceae | Grass |
| Pogonarthria squarrosa | Herringbone grass | Poaceae | Grass |
| Ehretia rigida | Sand paper bush | Boraginaceae | Tree |
| Terminalia sericea | Silver cluster-leaf | Combretaceae | Tree |
| Heteropogon contortus | Spear grass | Poaceae | Grass |
| Setaria sphacelata | Creeping bristle grass | Poaceae | Grass |
| Themeda triandra | Red oat grass | Poaceae | Grass |
| Urochloa mosambicensis | Bushveld signal grass | Poaceae | Grass |
| Vangueria infausta | Wild medlar | Rubiaceae | Shrub/tree |
| Setaria incrassate | Vlei bristle grass | Poaceae | Grass |
| Securinega virosa | White berry bush | Phyllanthaceae | Tree |
| Ximenia caffra | Sour plum | Olacaceae | Tree |
| Dalbergia melanoxylon | Zebra wood | Fabaceae | Tree |
| Ziziphus mucronata | Buffalo thorn | Rhamnaceae | Tree |
